# Identification of hub genes of Parkinson's disease through bioinformatics analysis

**DOI:** 10.3389/fnins.2022.974838

**Published:** 2022-11-08

**Authors:** Yajun Yang, Yi Wang, Ce Wang, Xinjuan Xu, Cai Liu, Xintao Huang

**Affiliations:** ^1^Department of Neurosurgery, The First Hospital of Shanxi Medical University, Taiyuan, China; ^2^The First School of Clinical Medicine, Shanxi Medical University, Taiyuan, China

**Keywords:** Parkinson's disease, identification, bioinformatics analysis, hub genes, GEO

## Abstract

Parkinson's disease (PD) is a common neurodegenerative disease, and there is still a lack of effective diagnostic and treatment methods. This study aimed to search for hub genes that might serve as diagnostic or therapeutic targets for PD. All the analysis was performed in R software. The expression profile data of PD (number: GSE7621) was acquired from the Gene Expression Omnibus (GEO) database. Differentially expressed genes (DEGs) associated with PD were screened by the “Limma” package of the R software. Key genes associated with PD were screened by the “WGCNA” package of the R software. Target genes were screened by merging the results of “Limma” and “WGCNA.” Enrichment analysis of target genes was performed by Gene Ontology (GO), Disease Ontology (DO), and Kyoto Enrichment of Genes and Genomes (KEGG). Machine learning algorithms were employed to screen for hub genes. Nomogram was constructed using the “rms” package. And the receiver operating characteristic curve (ROC) was plotted to detect and validate our prediction model sensitivity and specificity. Additional expression profile data of PD (number: GSE20141) was acquired from the GEO database to validate the nomogram. GSEA was used to determine the biological functions of the hub genes. Finally, *RPL3L, PLEK2, PYCRL, CD99P1, LOC100133130, MELK, LINC01101*, and *DLG3-AS1* were identified as hub genes of PD. These findings can provide a new direction for the diagnosis and treatment of PD.

## Introduction

Parkinson's disease (PD) is one of the common neurodegenerative diseases, second only to Alzheimer's disease (Di Stefano and Marinelli, 2021; Pan et al., 2021). The global prevalence of PD is rising, affecting nearly 2% of people over the age of 65 and 5% of people over the age of 85 (Bloem et al., 2021; Dorszewska et al., 2021). The pathological features of PD are mainly the loss of dopaminergic neurons in the substantia nigra and the formation of Lewy bodies, which is determined by genetic and environmental factors, and related to age, immune-inflammatory mechanisms, mitochondrial dysfunction, oxidative stress, apoptosis, lysosomal dysfunction, etc (Pan-Montojo and Reichmann, 2014; Su and Federoff, 2014; Kalia and Lang, 2015; Mullin and Schapira, 2015; Vivekanantham et al., 2015; Hu and Wang, 2016; Collier et al., 2017; Vascellari and Manzin, 2021). The role of genetic factors in PD is receiving more attention, and dozens of genes have been found to be related to the incidence of PD, including *SNCA, LRRK2*, and so on (Manto and Marmolino, 2009; Deng et al., 2018; Poujois and Woimant, 2018). Neurologists' assessments of clinical manifestations, movement disorders, and some routine laboratory tests are the most important diagnostic methods for PD. However, these methods have disadvantages and limitations, such as low sensitivity, selectivity, and high cost (Bindas et al., 2021; Mobed et al., 2021).

The treatment of PD is constantly developing, including drug therapy, surgical treatment, gene therapy, rehabilitation, etc. However, drug therapy is still the preferred treatment of Parkinson's disease in the clinic and is the main treatment method. However, drug therapy can only improve symptoms and cannot control the progression of the disease. With the prolongation of the medication duration and the dose increase, there will be a decrease in the efficacy of the drug and complications. Some researchers are developing new gene promoters to control gene expression in different subsets of neurons, stimulating the growth and development of specific neurons (such as dopaminergic neurons), thereby promoting the recovery of Parkinson's disease. Another emerging form of gene therapy is using novel drugs that directly deliver key proteins involved in dopamine metabolism to the basal ganglia region. Using gene therapies to clear disease-causing proteins is another beneficial exploration (Brundin et al., 1987; Lindvall et al., 1988; Ropper and Samuels, 1997). Although many genes are associated with PD, the specific pathogenesis is not clear, and there is still a lack of understanding of the genes that can be used for the diagnosis and treatment of PD. It is important to find new genes related to PD, which can be used for new targets for treatment.

Recently, bioinformatics analysis has been widely used as a new technique to screen for underlying biomarkers for both tumor and non-tumor diseases (Zhang et al., 2022). In this study, we downloaded a microarray dataset and analyzed gene expression to obtain differentially expressed genes from persons with PD and healthy individuals. We combined the data with WGCNA and machine learning algorithms, screened out a total of eight core genes, verified the prediction accuracy by area size (AUC) under the ROC curve, and performed GSEA analysis on each hub gene. We aimed to identify candidate genes that may be used as PD biomarkers.

## Materials and methods

### Data collection and preprocessing

The raw data of the substantia nigra tissue from 16 PD and nine American normal samples in the GSE7621 (Lesnick et al., 2007) dataset, which was sequenced using the GPL570 platform, was obtained from the GEO database.

### Identification of DEGs in the substantia nigra of patients with PD

The “Limma” R package was used to screen DEGs between PD and normal samples, and genes with *P* < 0.05 and |log_2_FC| >1 were regarded as DEGs (Liu et al., 2021).

### Screening of key modules and target genes based on WGCNA

To screen potential genes associated with PD, the gene expression matrix of the substantia nigra tissue from 16 PD and nine normal American samples was used to create a weighted gene co-expression network using the “WGCNA” R package (Langfelder and Horvath, 2008; Yu et al., 2012). First, we clustered all samples to guarantee a reliable network. Second, we calculated the Pearson correlation coefficient between each pair of genes to evaluate the expression similarity of genes and acquire a correlation matrix. We also used the soft threshold function to convert the correlation matrix into a weighted neighborhood matrix and used a soft connectivity algorithm to select the optimal soft threshold to ensure that gene correlations were maximally consistent with scale-free distribution. Subsequently, the neighborhood matrix was transformed into a topological overlap matrix (TOM). Furthermore, co-expression modules were obtained based on the criteria of dynamic tree cutting by setting the minimum number of genes in a module as 50. Finally, key modules were selected by correlation analysis, and the key modules' genes were considered key genes. Target genes were obtained by intersecting DEGs with key genes based on WGCNA screening.

### Gene Ontology, Disease Ontology, and Kyoto Enrichment of Genes and Genomes enrichment analyses

Biological function enrichment of Gene Ontology (GO), Disease Ontology (DO), and Kyoto Enrichment of Genes and Genomes (KEGG) analyses were performed using the “clusterProfiler” R (Yu et al., 2012) package. GO enrichment analysis was performed to investigate the gene-related biological process (BP), molecular functions (MF), and cellular components (CC). DO enrichment analysis was used to explore genes-related diseases. KEGG enrichment analysis was conducted to explore gene-related signaling pathways. Statistical significance was set at an adjusted *P*-value < 0.05.

### Identification of hub genes of PD based on machine learning algorithms

To begin with, the LASSO logistic regression algorithm was performed to screen potential genes by using the “glmnet” R package (Tibshirani, 1996; Friedman et al., 2010), and receiver operating characteristic (ROC) analysis was selected to test the model reliability by calculating the area under the curve (AUC) value through the “pROC” R package (Robin et al., 2011). Next, the SVM-RFE algorithm was used to screen potential genes using the “e1071” R package (Suykens and Vandewalle, 2004; Huang et al., 2014). In addition, the random forest (RF) algorithm was also conducted to screen potential genes using the “randomForest” R package (Liaw and Wiener, 2007; Cutler et al., 2011). Similarly, the ROC curve was used to test the model reliability by using the “pROC” R package, and the top 10 genes based on %IncMSE ranking were regarded as potential genes (Robin et al., 2011). Finally, overlapping genes among potential genes generated via LASSO, SVM-RFE, and RF algorithms were considered hub genes of PD.

### Establishment of a diagnostic nomogram for PD

A diagnostic nomogram was established based on the hub genes by using the “rms” package in R software. The receiver operating characteristic curve (ROC) was used to investigate the efficiency of this diagnostic model. The area under curve >0.7 was considered significant. Additional expression profile data of PD [number: GSE20141 (Zheng et al., 2010)] was acquired from the GEO database to validate the nomogram.

### Evaluation of the expression levels and diagnostic implications for the hub genes

Wilcoxon's rank-sum test was used to analyze the expression levels of hub genes. ROC analysis was performed to evaluate whether hub genes could differentiate PD samples from normal samples using the “pROC” R package (Robin et al., 2011).

### Biological functions and validation of hub genes

Gene Set Enrichment Analysis (GSEA) was performed using the “clusterProfiler” R package to investigate the biological functions of hub genes by the ordered gene expression matrix based on the Pearson correlation between each hub gene and other genes (Yu et al., 2012).

## Results

### Identification of DEGs in the substantia nigra of patients with PD

**Figure 1:**
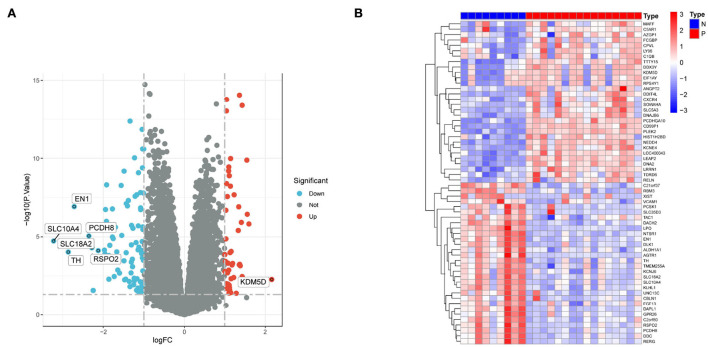
DEGs between PD patients and normal samples. **(A)** Volcano plot showing the expression levels of DEGs. Red dots indicate upregulated genes in PD patients compared with normal samples. In contrast, blue dots indicate downregulated genes in PD patients compared with normal samples, and gray dots indicate nonsignificant differences in genes between PD patients and normal samples. **(B)** Heat map showing the expression levels of the top 60 DEGs. Red indicates high expression, while blue indicates low expression.

By setting the cut-off value as *P* < 0.05 and |log_2_FC| >1, a total of 117 DEGs, including 42 upregulated and 75 downregulated genes were identified in the substantia nigra of PD patients compared with normal samples (Table 1). A volcano diagram was constructed for the DEGs (Figure 1A). The top 60 DEGs are presented using a cluster heatmap (Figure 1B).

**Table 1:** The DEGs of gene expression profiles (adj. *P*-value <0.05, |logFC| >1.0).

**DEGs**	**Gene symbol**
Upregulated DEGs	*CD99Pl;RPL3L;PLEK2;RAB42;DLG3-AS1;LlNC01101;lL13;MELK;PYCRL;L EAP2;DNA2;PCDHGA8;C15orf37;LOC100288893; LPO;LOC100133130;NEDD4;LOC100130987;DACH2; NTSR1;PCDHGA1O;ASIC2;lNSM2;SNORD114-3; LOC400043;RBM11;LlNC01158;CNTN6;CCT6B; LOC441052;EN1;TTTY15;ABCA11P;DLK1;C21orf37; WDR17;PIM1;KCNE4;DAPLl;LRRN1;DDIT4L;SDCl*
Downregulated DEGs	*SSTR1;LINC00515;RBM3;MAPK8IP1;KDR;DNAJB6; RERG;PCDH8;KLHL1;AGTR1;TNRC6C-AS1;SLC10A4; CUX2;EBF3;TlMM238;DDC;NANOS1;SLC18A2;PRMT6; ALDH1A1;KCNE1L;RSP02;SLC5A3;SPA17;C2orf80; TH;HIST1H2BD;GBE1;C5orf64;UNC13C;UHRF1; TMEM255A;SDR16C5;ROBO2;CLSTN2;CDH8; GPR26;MID1IP1;KCNJ6;RET;HMOX1;SOWAHA; DDX3Y;RELN;TDRD6;CPVL;NR4A2;PCSK1;AKR1C3; GABRA4;BCL6;RHOBTB1;RNASE2;PSPH;LRRC3B; VCAM1;TAC1;C5AR1;ANGPT2;DOK6;CTXN3;CXCR4; SLC35D3;FGF13;KDM5D;CBLN1;FGF12;OLFM3;APLNR; COPG2IT1;S100A4;FZD7;OPALlN;LY96;ELAVL2*

### Screening of key modules and genes based on WGCNA

**Figure 2:**
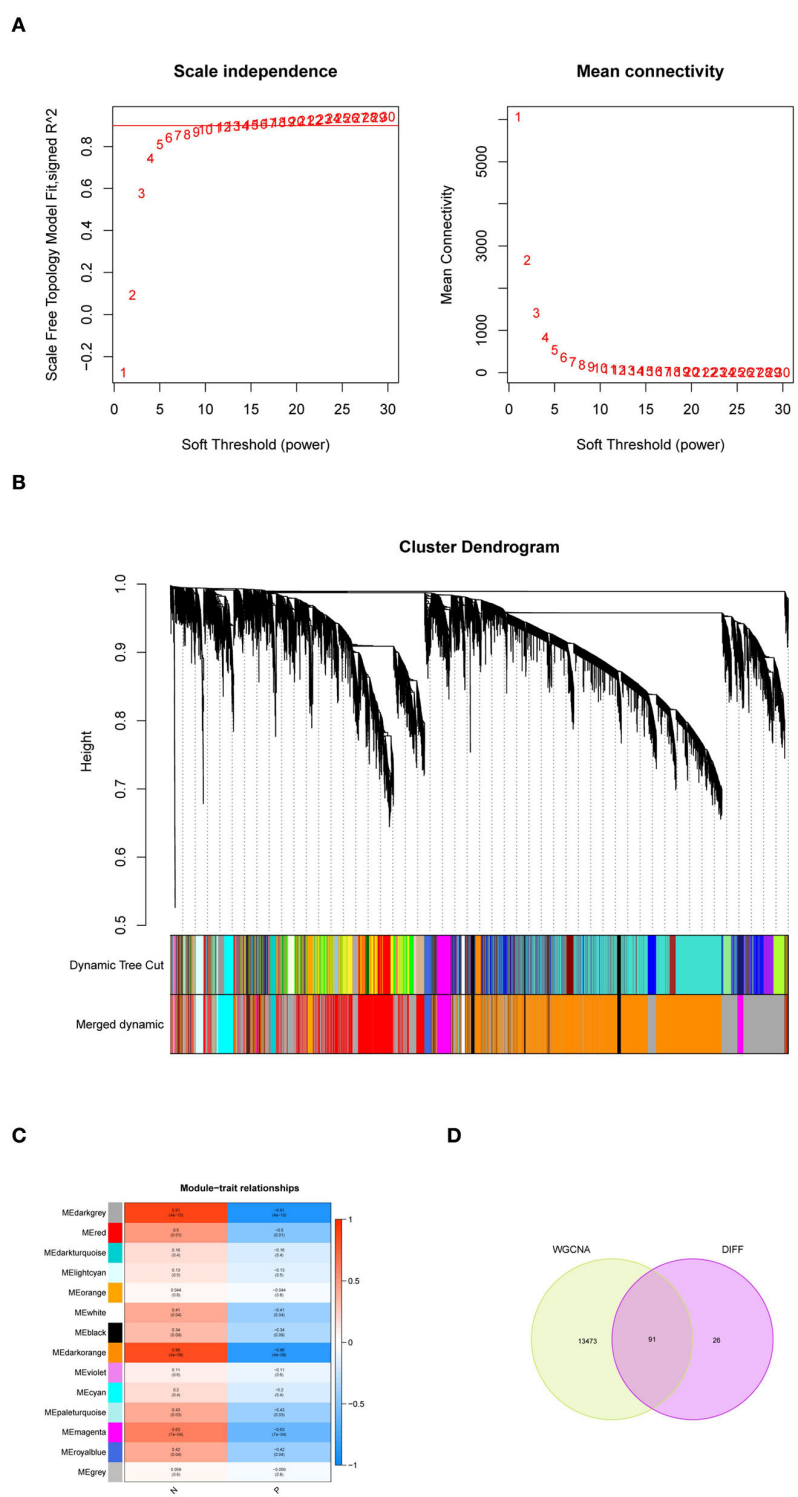
Weighted gene co-expression network analysis (WGCNA). **(A)** Soft threshold analysis suggested gene associations were maximally consistent with the scale-free distribution when β = 26. **(B)** The cluster dendrogram of co-expression genes in PD. **(C)** Module-trait relationships in PD. Each cell contains the corresponding correlation and *P*-value. **(D)** A Venn diagram was made to obtain the intersection of the target genes screened by the two methods.

We extracted the expression data of differentially expressed genes in samples of persons with PD for co-expression analysis. First, the soft threshold was selected for subsequent co-expression network construction (Figure 2A). The principle was to make the constructed network more in line with the characteristics of the scale-free network. The *R*-square was set as 0.85 (Figure 2A). WGCNA was used to construct the co-expression network module and visually display the modules' gene correlation. Fourteen co-expression modules were obtained, and the number of genes in each module was at least 50. The results were displayed in a hierarchical clustering diagram (Figure 2B). Then, a heat map was mapped on module-trait relationships according to the Spearman correlation coefficient to evaluate the association between each module and the disease (Figure 2C). Two modules “MEdarkgrey” and “MEdarkorange” had high association with PD and were selected as PD-related modules (MEdarkgrey module: *r* = 0.91, *P* = 4e−10; MEdarkorange module: *r* = 0.89, *P* = 4e−08). The MEdarkgrey and MEdarkorange modules were positively correlated with PD, 5,801, and 7,763 genes, respectively. Target genes were obtained by intersecting DEGs with key genes based on WGCNA screening (Figure 2D).

### Gene Ontology, Disease Ontology, and Kyoto Encyclopedia of Genes and Genomes enrichment analyses

**Figure 3:**
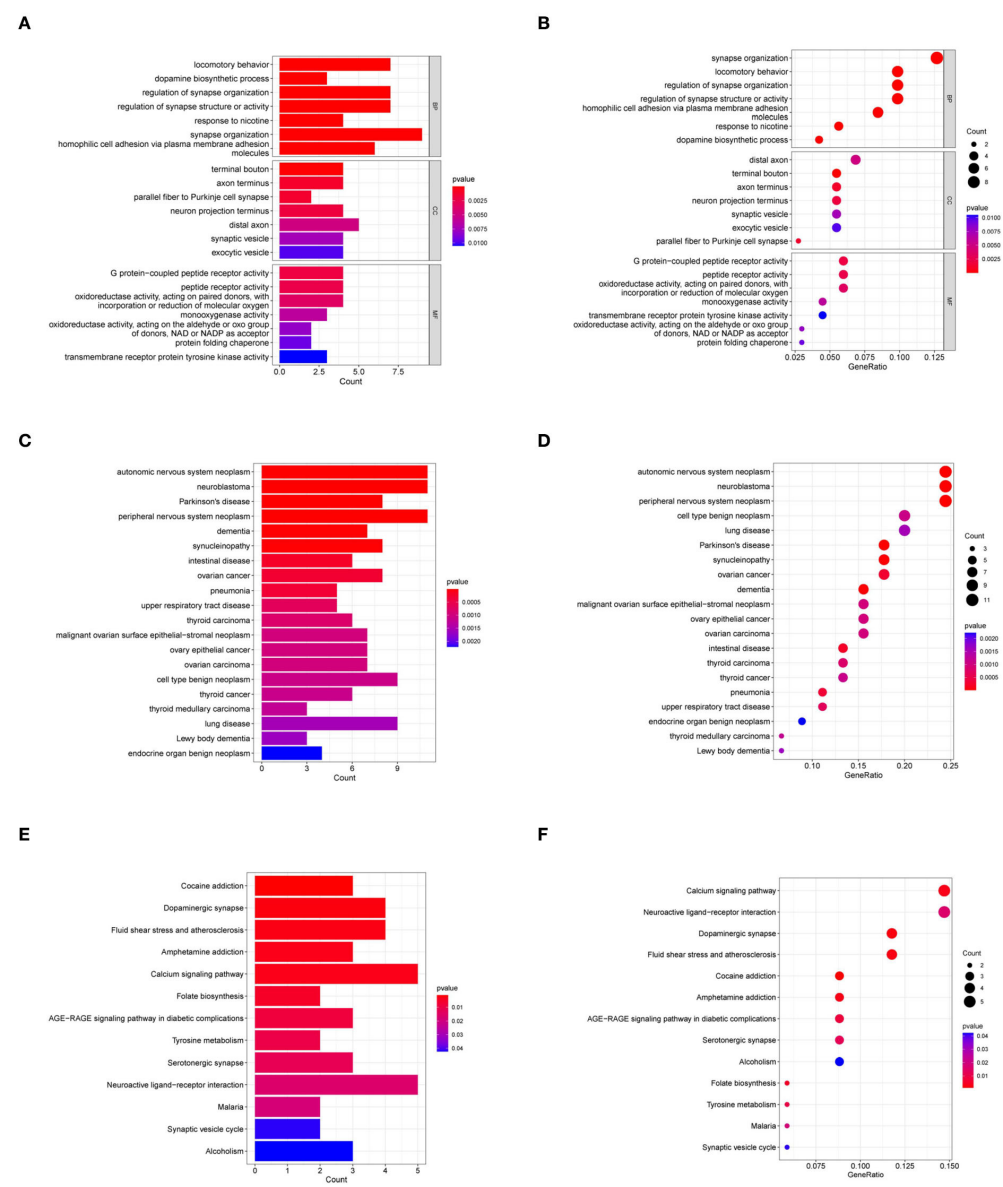
GO, DO, and KEGG analyses of target genes. **(A,B)** GO analyses of target genes. **(C,D)** DO analysis of target genes. **(E,F)** KEGG analysis of target genes. BP, biological process; CC, cellular component; MF, molecular function.

The GO analyses associated the most enriched biological process (BP) terms with dopamine biosynthetic process, synapse organization regulation, and synapse structure or activity. The most enriched terms for cellular components (CC) were mainly associated with terminal bouton. The most enriched molecular function (MF) terms were associated with G protein-coupled peptide receptor activity and peptide receptor activity (Figures 3A,B). In the DO analysis, the target genes were enriched in neurodegenerative diseases, such as PD, and tumors of the nervous system (Figures 3C,D). In the KEGG analysis, the target genes were enriched in cocaine addiction and the calcium signaling pathway (Figures 3E,F).

### Identification of hub genes of PD based on machine learning algorithms

**Figure 4:**
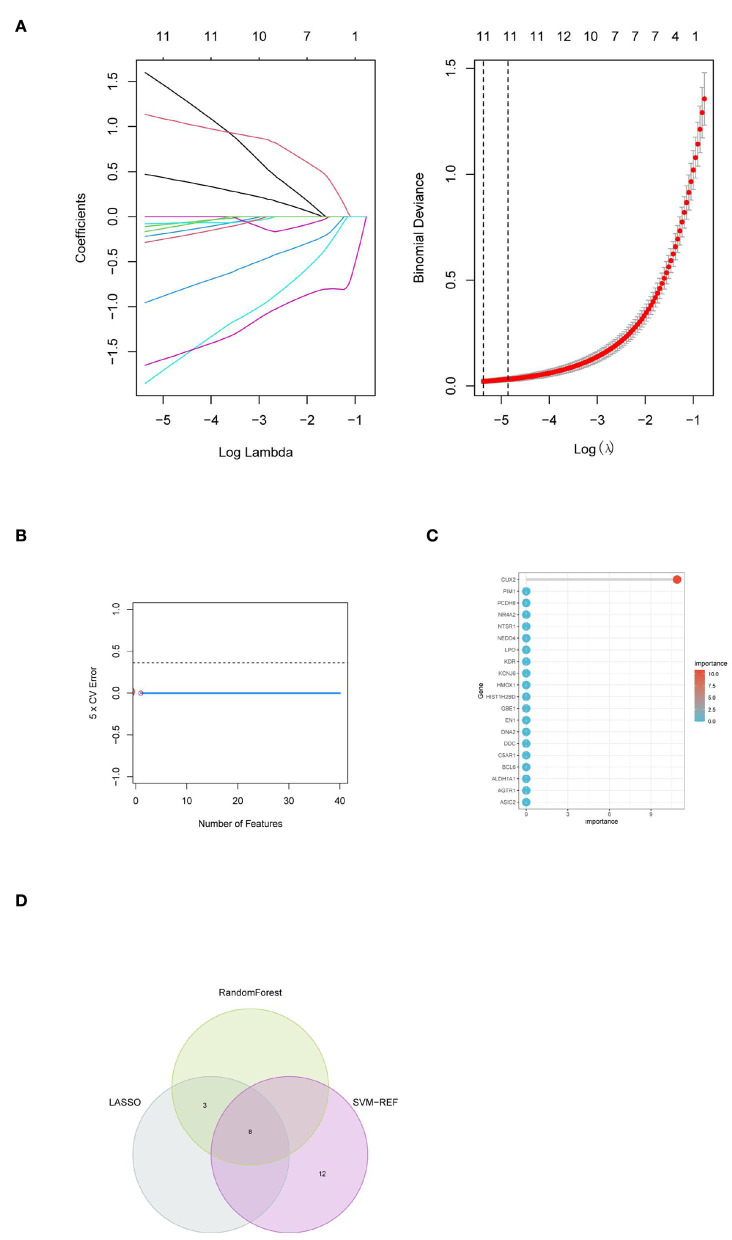
Identification of hub genes for PD based on machine learning algorithms included **(A)** the Log (Lambda) value of the three genes in the LASSO model and the most proper log (Lambda) value in the LASSO model, **(B)** the optimum error rate of the SVM model based on one characteristic gene, **(C)** the RF module based on the top 20 genes, and **(D)** the Venn diagram showing the overlapping genes in LASSO, SVM, and RF modules.

**Figure 5:**
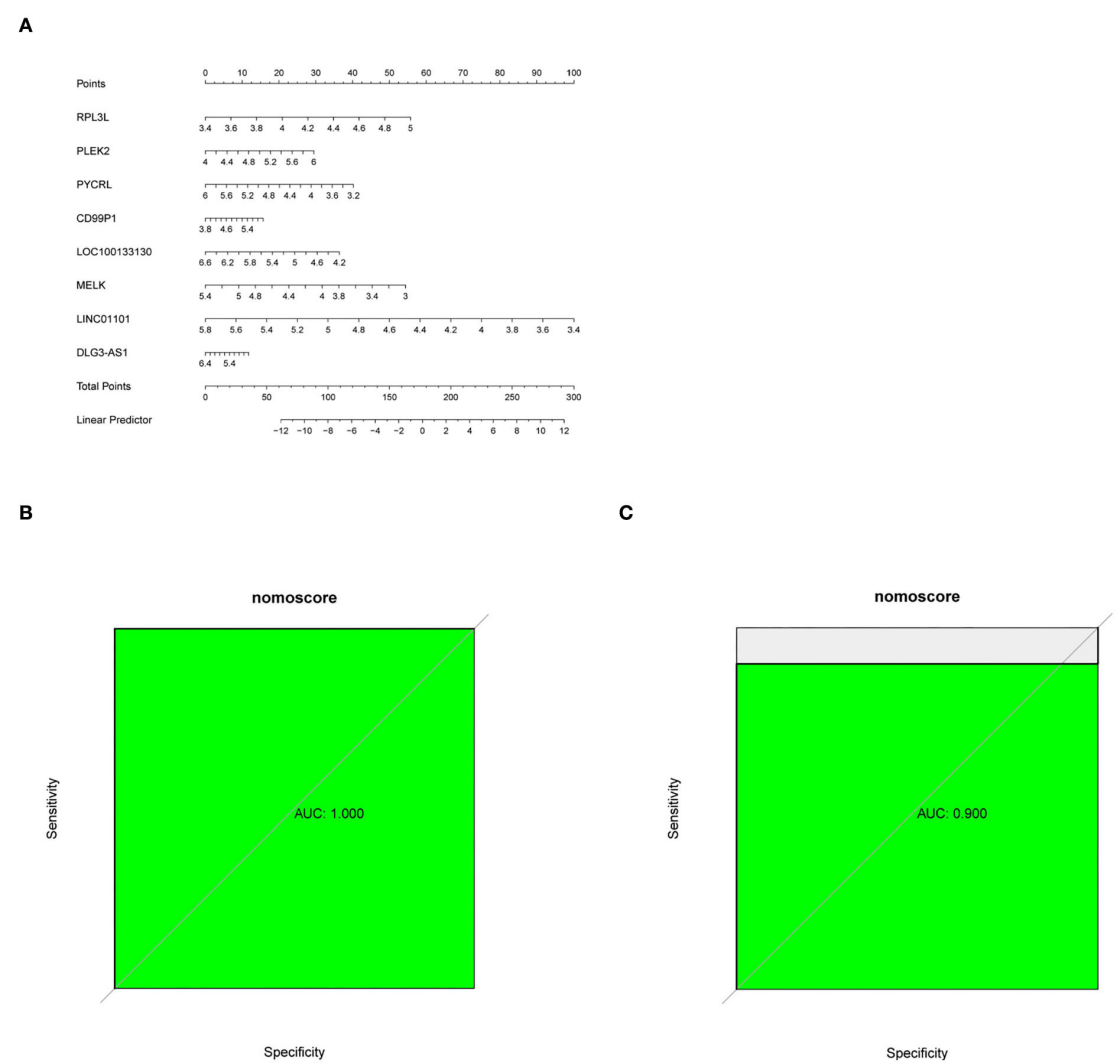
**(A)** Nomogram predicting PD probability. **(B,C)** ROC curve for the GSE7621 dataset and GSE20141 dataset.

Machine learning algorithms were selected and executed to further identify the hub genes of PD from 91 target genes. First, while constructing the LASSO model based on PD and normal samples, λ analysis suggested that the model could accurately predict PD with λ = 11 (Figure 4A). Thus, *RPL3L, PLEK2, PYCRL, ABCA11P, DACH2, CD99P1, SNORD114-3, LOC100133130, MELK, LINC01101*, and *DLG3-AS1* were identified to build the LASSO module. We acquired the LASSO coefficient spectrum of the potential genes according to λ = 11(Figure 4A). However, SVM-RFE analysis revealed that the SVM model based on one characteristic gene showed an optimum error rate (0.00, Figure 4B). The first 20 genes were identified as potential genes. At the same time, the RF algorithm identified the top 10 genes from 52 potential genes (Figure 5C). Finally, eight common potential genes, namely, *RPL3L, PLEK2, PYCRL, CD99P1, LOC100133130, MELK, LINC01101*, and *DLG3-AS1*, were regarded as the hub genes of PD patients using the above three algorithms (Figure 4D).

### Establishment of diagnostic nomogram for PD

A diagnostic nomogram was successfully constructed based on the eight genes for predicting the incidence of PD (Figure 5A). The area under the curve (AUC) of the GSE7621 dataset was 1.000 (Figure 5B), and that of the GSE20141 dataset was 0.900 (Figure 5C).

### Evaluation of the expression levels and diagnostic implications for the hub genes

**Figure 6:**
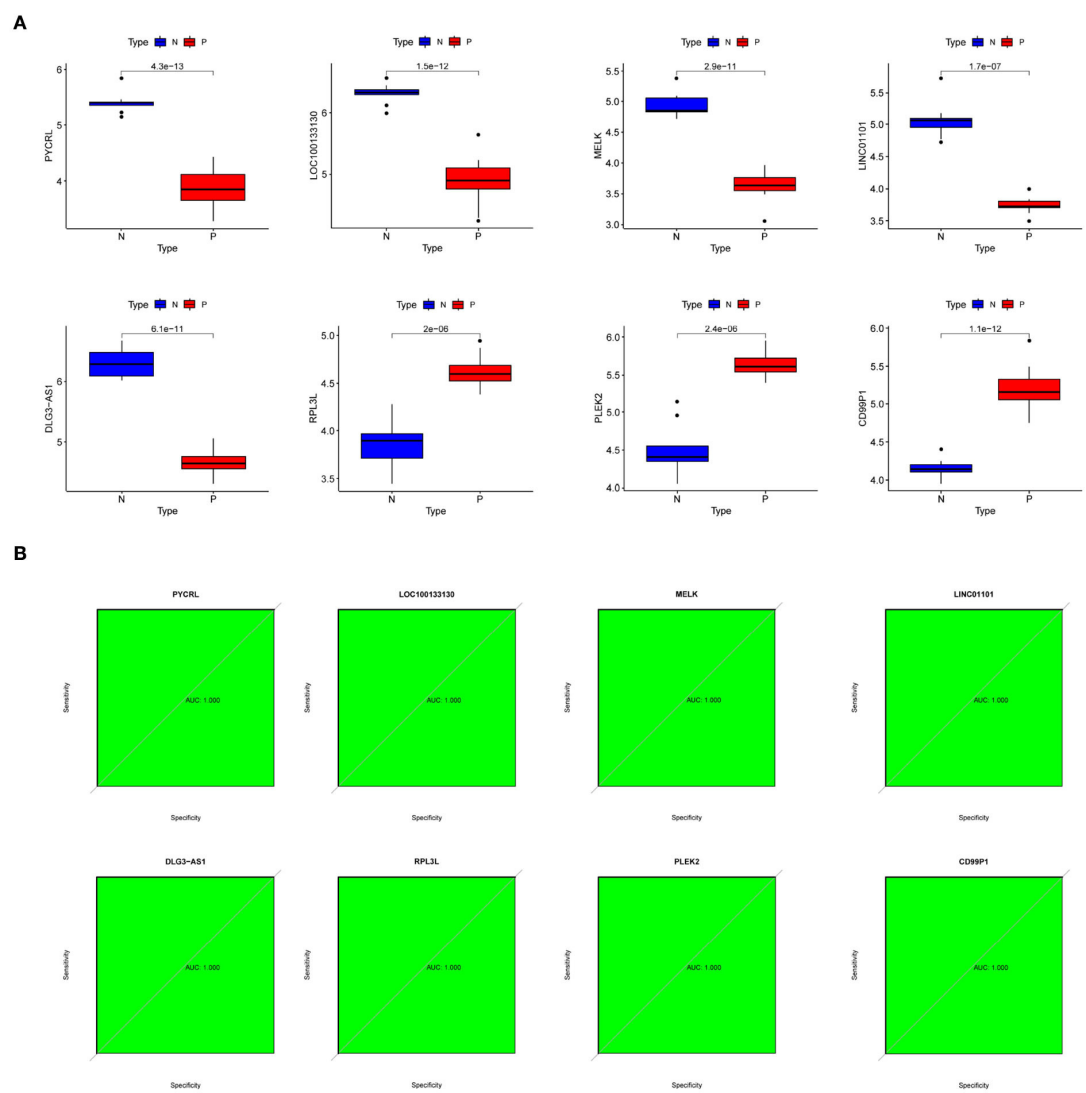
The expression levels and diagnostic implications of the hub genes. **(A)** The expression levels of hub genes in PD patients and normal samples of the dataset (number: GSE7621); **(B)** ROC curves of hub genes of the dataset (number: GSE7621).

To further investigate the role of hub genes in PD, we first observed their expression levels in PD patients. Interestingly, we found that the expression of *PYCRL, LOC100133130, MELK, LINC01101*, and *DLG3-AS1* was downregulated, and the expression of *RPL3L, PLEK2*, and *CD99P1* was upregulated in PD patients compared with the healthy samples (Figure 6A). Moreover, ROC analyses suggested *RPL3L, PLEK2, PYCRL, CD99P1, LOC100133130, MELK, LINC01101*, and *DLG3-AS1* might be used as hub genes of PD patients (Figure 6B).

### Biological functions of hub genes

**Figure 7:**
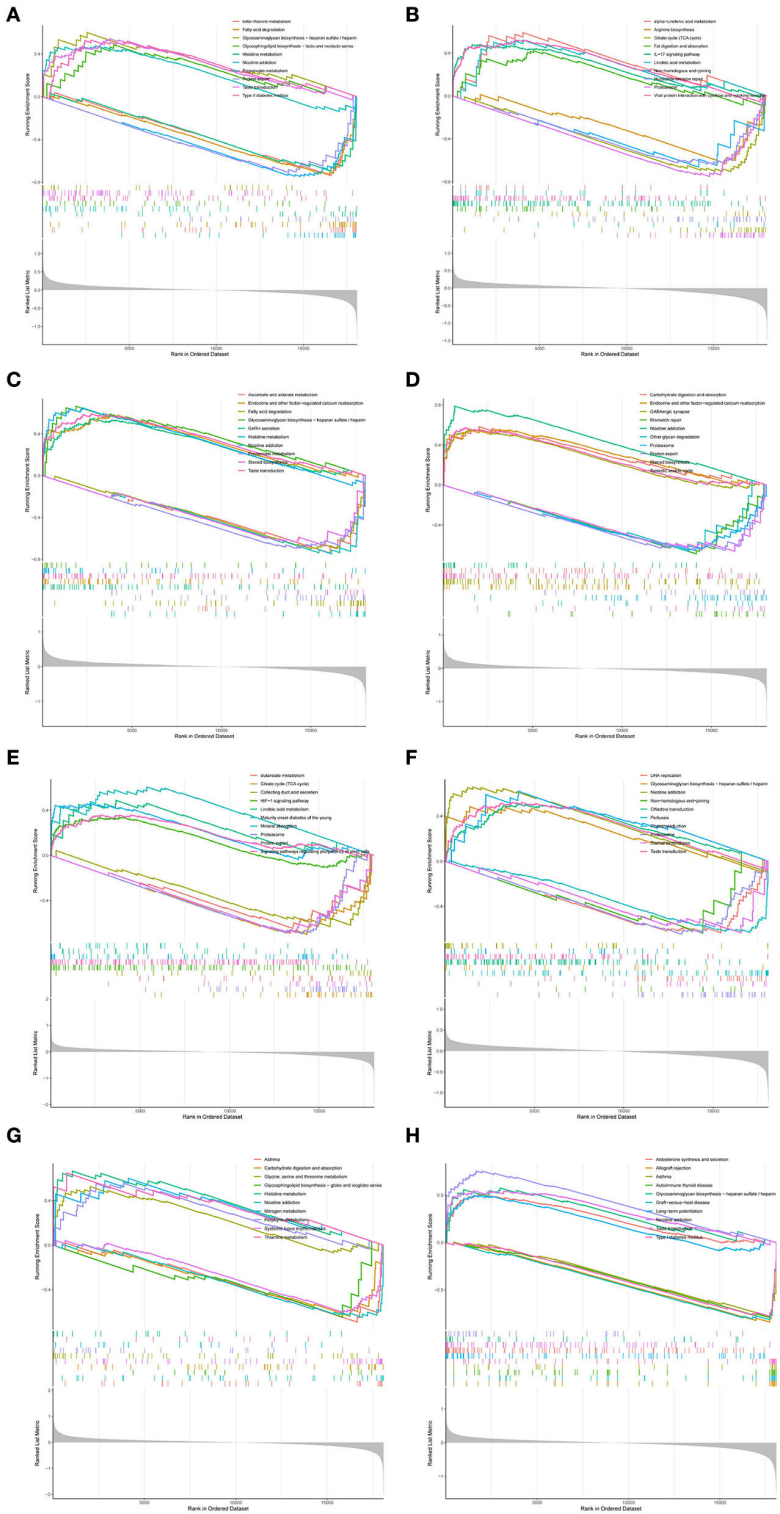
GSEA of hub genes: **(A)** GSEA results for *CD99P1*; **(B)** GSEA results for *DLG3-AS1*; **(C)** GSEA results for *LINC01101*; **(D)** GSEA results for *LOC100133130*; **(E)** GSEA results for *MELK*; **(F)** GSEA results for *PLEAK2*; **(G)** GSEA results for *PYCRL*; **(H)** GSEA results for *RPL3L*.

To further investigate the biological functions of *RPL3L, PLEK2, PYCRL, CD99P1, LOC100133130, MELK, LINC01101*, and *DLG3-AS1*, GSEA was performed based on their ordered gene expression matrix. As shown in Figure 7, GSEA analyses revealed that *RPL3L, PLEK2, PYCRL, CD99P1, LOC100133130, MELK, LINC01101*, and *DLG3-AS1* were mainly involved in glycosaminoglycan biosynthesis – heparan sulfate/heparin, histidine metabolism, nicotine addiction, diabetes, protein export, taste transduction, fatty acid degradation, propanoate metabolism, endocrine, and other factor–regulated calcium reabsorption, GABAergic synapse, steroid biosynthesis, synaptic vesicle cycle, carbohydrate digestion and absorption, proteasome, and asthma (Figure 7).

## Discussion

Parkinson's disease is the second most prevalent neurodegenerative disease and has significantly increased over the past 20 years (Cabreira and Massano, 2019). It is caused by a combination of environmental and genetic factors related to age, sex, etc (Cabreira and Massano, 2019; Cerri et al., 2019). Due to the lack of early diagnostic techniques, PD is usually not detected until later stages of complete neuronal degeneration, which often leads to delayed treatment of patients and affects the prognosis (Lotankar et al., 2017). Therefore, to improve prognosis, biomarkers are needed to detect the onset of the disease in the early stages.

In the present study, we first obtained 117 DEGs from the substantia nigra of PD, and WGCNA screened a total of 13,564 key genes for two key modules. Ninety-one target genes were screened out by intersecting DEGs with key genes. Interestingly, these 91 target genes were mostly related to the regulation of synapse structure or activity, dopamine biosynthetic process, locomotory behavior, and response to nicotine metabolism-related BPs (Figures 3A,B). Thus, we speculated that these genes might play key roles in PD by regulating the synapse structure or activity, dopamine biosynthetic process, locomotory behavior, and response to nicotine.

Over the past few decades, more and more studies have shown that the biosynthesis of dopamine and the regulation of synaptic activity and structure play an important role in the pathogenesis of PD (Latif et al., 2021; Nachman and Verstreken, 2022). In addition, increasing evidence has revealed that nicotine has protective effects on PD (Jin Jung et al., 2021; Wang et al., 2022). Furthermore, motor symptoms are one of the most important clinical manifestations of PD (Opara et al., 2017). However, we found that 91 target genes were enriched only in the calcium signaling pathway (Figures 3E,F). Calcium signaling pathways play an important role in PD (Calì et al., 2014; Bohush et al., 2021). Therefore, our study may contribute to understanding the molecular mechanisms underlying PD.

Finally, we identified *RPL3L, PLEK2, PYCRL, CD99P1, LOC100133130, MELK, LINC01101*, and *DLG3-AS1* as hub genes using LASSO logistic regression, SVM-RFE, and RF algorithms. *RPL3L* (ribosomal protein L3-like) is one of the four non-canonical riboprotein genes, and it encodes the 60S ribosomal protein L3-like protein that is highly expressed only in cardiac and skeletal muscles (Ganapathi et al., 2020). Recent research indicates that *RPl3l* overexpression impairs the growth and myogenic fusion of myotubes, and *RPL3L* can be used as a potential genetic marker to control neurodegeneration (Chaillou, 2019). Thus, RPL3L may play a critical role in PD by regulating function of skeletal muscle. *CD99P1* (CD99 molecule pseudogene 1) is a long noncoding RNA (lncRNA) and has been revealed to be related to myofibroblast differentiation (Huang et al., 2015; Yildirim et al., 2021). Hence, *CD99P1* may play a key role in PD by affecting myofibroblast differentiation. *PYCRL* (pyrroline-5-carboxylate reductase-like) is a pyrroline-5-carboxylate reductase linked to the conversion of ornithine to proline (De Ingeniis et al., 2012). Therefore, *PYCRL* may play a decisive role in PD by affecting proline biosynthesis. *MELK* (maternal embryonic leucine zipper kinase) is an AMP-activated protein kinase (AMPK)-related kinase (Seong and Ha, 2019). More and more studies have found that *MELK* is involved in the occurrence of cancer and cell metabolism by regulating the cell division cycle (Wang et al., 2018; Seong and Ha, 2019). Therefore, *MELK* may play a key role in PD by affecting the cell division cycle. *PLEK2* (Pleckstrin-2) is a crucial mediator of cytoskeletal reorganization (Wang et al., 2021). Studies show that *PLEK2* may regulate actin organization and cell spreading (Hu et al., 1999; Hamaguchi et al., 2007). Therefore, *PLEK2* may play a key role in PD by regulating actin organization and cell spreading. *LINC01101* is a long noncoding RNA (lncRNA) associated with progression and high-risk HPV infection (Iancu et al., 2017). But there is still no description of its pathogenesis. Notably, no studies have reported the role of *RPL3L, PLEK2, PYCRL, CD99P1, LOC100133130, MELK, LINC01101*, and *DLG3-AS1* in PD. *LOC100133130* and *DLG3-AS1*, as newly discovered genes, have no relevant report. Thus, further investigations are necessary.

We investigated the biological functions of *RPL3L, PLEK2, PYCRL, CD99P1, LOC100133130, MELK, LINC01101*, and *DLG3-AS1*. Interestingly, GSEA revealed that *RPL3L, PLEK2, PYCRL, CD99P1, LOC100133130, MELK, LINC01101*, and *DLG3-AS1* were mainly involved in nicotine addiction, diabetes, protein export, taste transduction, fatty acid degradation, propanoate metabolism, endocrine and other factor-regulated calcium reabsorption, GABAergic synapse, steroid biosynthesis, synaptic vesicle cycle, carbohydrate digestion and absorption, proteasome, and asthma. Currently, an increasing number of studies have shown that the above factors significantly impact the occurrence and development of PD. For example, it has been suggested that nicotine addiction and diabetes are associated with PD (Cheong et al., 2020; Carvajal-Oliveros et al., 2021). However, their regulatory mechanisms are rarely studied to the best of our knowledge. Thus, further studies are required to explore this in the future.

## Conclusion

In all, 117 DEGs were screened between the substantia nigra of PD and healthy samples. Of these, *RPL3L, PLEK2, PYCRL, CD99P1, LOC100133130, MELK, LINC01101*, and *DLG3-AS1* were identified as hub genes of patients with PD based on WGCNA and machine learning algorithms. Therefore, our study contributes to the understanding of PD and helps in improving the diagnosis and treatment of PD. However, further studies are needed to investigate the roles of hub genes.

## Data availability statement

The original contributions presented in the study are included in the article/supplementary material, further inquiries can be directed to the corresponding author.

## Ethics statement

Ethical review and approval was not required for the study on human participants in accordance with the local legislation and institutional requirements. Written informed consent from the patients/participants or patients/participants' legal guardian/next of kin was not required to participate in this study in accordance with the national legislation and the institutional requirements.

## Author contributions

YY conceived of the presented idea. XH developed the theory, performed the computations, and supervised the findings of this work. CW and YW verified the analytical methods. XX and CL contributed in the revision of the article. All authors discussed the results and contributed to the final manuscript.

## Funding

This work was funded by Research Project Supported by Shanxi Scholarship Council of China (2020-169), Fund Program for the Scientific Activities of Selected Returned Overseas Professionals in Shanxi Province (20210023), and Fundamental Research Program of Shanxi Province (202203021211017).

## Conflict of interest

The authors declare that the research was conducted in the absence of any commercial or financial relationships that could be construed as a potential conflict of interest.

## Publisher's note

All claims expressed in this article are solely those of the authors and do not necessarily represent those of their affiliated organizations, or those of the publisher, the editors and the reviewers. Any product that may be evaluated in this article, or claim that may be made by its manufacturer, is not guaranteed or endorsed by the publisher.

## References

[B1] Bindas A. J. Kulkarni S. Koppes R. A. Koppes A. N. (2021). Parkinson's disease and the gut: models of an emerging relationship. Acta Biomater. 132, 325–344. 10.1016/j.actbio.2021.03.071 33857691

[B2] Bloem B. R. Okun M. S. Klein C. (2021). Parkinson's disease. Lancet. 397, 2284–2303. 10.1016/S0140-6736(21)00218-X 33848468

[B3] Bohush A. Leśniak W. Weis S. Filipek A. (2021). Calmodulin and its binding proteins in Parkinson's disease. Int. J. Mol. Sci. 22. 10.3390/ijms22063016 33809535 PMC8001340

[B4] Brundin P. Strecker R. E. Lindvall O. Isacson O. Nilsson O. G. Barbin G.. (1987). Intracerebral grafting of dopamine neurons. Experimental basis for clinical trials in patients with Parkinson's disease. Ann. N. Y. Acad. Sci. 495, 473–96. 10.1111/j.1749-6632.1987.tb23695.x 3474955

[B5] Cabreira V. Massano J. J. A. M. P. (2019). Doença de Parkinson: revisão clínica e atualização. Parkinson's disease: clinical review and update. Acta. Med. Port. 32:661–670. 10.20344/amp.11978 31625879

[B6] Calì T. Ottolini D. Brini M. (2014). Calcium signaling in Parkinson's disease. Cell Tissue Res. 357, 439–54. 10.1007/s00441-014-1866-0 24781149

[B7] Carvajal-Oliveros A. Domínguez-Baleón C. Zárate R. V. Campusano J. M. Narváez-Padilla V. Reynaud E.. (2021). Nicotine suppresses Parkinson's disease like phenotypes induced by Synphilin-1 overexpression in *Drosophila melanogaster* by increasing tyrosine hydroxylase and dopamine levels. Sci. Rep. 11, 9579. 10.1038/s41598-021-88910-4 33953275 PMC8099903

[B8] Cerri S. Mus L. Blandini F. (2019). Parkinson's disease in women and men: what's the difference? J. Parkinsons Dis. 9, 501–515. 10.3233/JPD-191683 31282427 PMC6700650

[B9] Chaillou T. (2019). Ribosome specialization and its potential role in the control of protein translation and skeletal muscle size. J. Appl. Physiol. 127, 599–607. 10.1152/japplphysiol.00946.2018 30605395

[B10] Cheong J. L. Y. de Pablo-Fernandez E. Foltynie T. Noyce A. J. (2020). The association between type 2 diabetes mellitus and Parkinson's disease. J. Parkinsons Dis. 10, 775–789. 10.3233/JPD-191900 32333549 PMC7458510

[B11] Collier T. J. Kanaan N. M. Kordower J. H. (2017). Aging and Parkinson's disease: different sides of the same coin? Mov. Disord. 32, 983–990. 10.1002/mds.27037 28520211 PMC5844262

[B12] Cutler A. Cutler D. Stevens J. (2011). “Random forests,” in Ensemble Machine Learning, eds C. Zhang and Y. Q. Ma, Vol. 45 (New York, NY: Springer), 157–176. 10.1007/978-1-4419-9326-7_5

[B13] De Ingeniis J. Ratnikov B. Richardson A. D. Scott D. A. Aza-Blanc P.. (2012). Functional specialization in proline biosynthesis of melanoma. PLoS ONE. 7, e45190. 10.1371/journal.pone.0045190 23024808 PMC3443215

[B14] Deng H. Wang P. Jankovic J. (2018). The genetics of Parkinson disease. Ageing Res. Rev. 42, 72–85. 10.1016/j.arr.2017.12.007 29288112

[B15] Di Stefano A. Marinelli L. (2021). Advances in Parkinson's disease drugs. Biomolecules. (2021) 11. 10.3390/biom11111640 34827638 PMC8615848

[B16] Dorszewska J. Kowalska M. Prendecki M. Piekut T. Kozłowska J. Kozubski W.. (2021). Oxidative stress factors in Parkinson's disease. Neural Regen. Res. 16, 1383–1391. 10.4103/1673-5374.300980 33318422 PMC8284265

[B17] Friedman J. Hastie T. Tibshirani R. (2010). Regularization paths for generalized linear models via coordinate descent. J. Stat. Softw. 33, 1–22. 10.18637/jss.v033.i01 20808728 PMC2929880

[B18] Ganapathi M. Argyriou L. Martínez-Azorín F. Morlot S. Yigit G. Lee T. M.. (2020). Bi-allelic missense disease-causing variants in RPL3L associate neonatal dilated cardiomyopathy with muscle-specific ribosome biogenesis. Hum. Genet. 139, 1443–1454. 10.1007/s00439-020-02188-6 32514796 PMC7519902

[B19] Hamaguchi N. Ihara S. Ohdaira T. Nagano H. Iwamatsu A. Tachikawa H.. (2007). Pleckstrin-2 selectively interacts with phosphatidylinositol 3-kinase lipid products and regulates actin organization and cell spreading. Biochem. Biophys. Res. Commun. 361, 270–5. 10.1016/j.bbrc.2007.06.132 17658464

[B20] Hu M. H. Bauman E. M. Roll R. L. Yeilding N. Abrams C. S. (1999). Pleckstrin 2, a widely expressed paralog of pleckstrin involved in actin rearrangement. J. Biol. Chem. 274, 21515–8. 10.1074/jbc.274.31.21515 10419454

[B21] Hu Q. Wang G. (2016). Mitochondrial dysfunction in Parkinson's disease. Transl. Neurodegener. 5, 14. 10.1186/s40035-016-0060-6 27453777 PMC4957882

[B22] Huang C. Yang Y. Liu L. (2015). Interaction of long noncoding RNAs and microRNAs in the pathogenesis of idiopathic pulmonary fibrosis. Physiol. Genomics. 47, 463–9. 10.1152/physiolgenomics.00064.2015 26269497 PMC4593830

[B23] Huang M. L. Hung Y. H. Lee W. M. Li R. K. Jiang B. R. (2014). SVM-RFE based feature selection and Taguchi parameters optimization for multiclass SVM classifier. ScientificWorldJournal. 2014, 795624. 10.1155/2014/795624 25295306 PMC4175386

[B24] Iancu I. V. Anton G. Botezatu A. Huica I. Nastase A. Socolov D. G.. (2017). LINC01101 and LINC00277 expression levels as novel factors in HPV-induced cervical neoplasia. J. Cell. Mol. Med. 21, 3787–3794. 10.1111/jcmm.13288 28767188 PMC5706512

[B25] Jin Jung Y. Choi H. Oh E. (2021). Effects of particulate matter and nicotine for the MPP+-induced SH-SY5Y cells: implication for Parkinson's disease. Neurosci. Lett. 765, 136265. 10.1016/j.neulet.2021.136265 34563623

[B26] Kalia L. V. Lang A. E. (2015). Parkinson's disease. Lancet. 386, 896–912. 10.1016/S0140-6736(14)61393-3 25904081

[B27] Langfelder P. Horvath S. (2008). WGCNA: an R package for weighted correlation network analysis. BMC Bioinformatics 9, 559. 10.1186/1471-2105-9-559 19114008 PMC2631488

[B28] Latif S. Jahangeer M. Maknoon Razia D. Ashiq M. Ghaffar A. Akram M.. (2021). Dopamine in Parkinson's disease. Clin. Chim. Acta. 522, 114–126. 10.1016/j.cca.2021.08.009 34389279

[B29] Lesnick T. G. Papapetropoulos S. Mash D. C. Ffrench-Mullen J. Shehadeh L. de Andrade M.. (2007). A genomic pathway approach to a complex disease: axon guidance and Parkinson disease. PLoS Genet. 3, e98. 10.1371/journal.pgen.0030098 17571925 PMC1904362

[B30] Liaw A. Wiener M. C. (2007). In *Classification and Regression by randomForest*.

[B31] Lindvall O. Rehncrona S. Gustavii B. Brundin P. Astedt B. Widner H.. (1988). Fetal dopamine-rich mesencephalic grafts in Parkinson's disease. Lancet 2, 1483–4. 10.1016/S0140-6736(88)90950-6 2904587

[B32] Liu D. Fan Y. B. Tao X. H. Pan W. L. Wu Y. X. Wang X. H.. (2021). Mitochondrial quality control in sarcopenia: Updated overview of mechanisms and interventions. Aging Dis. 12, 2016–2030. 10.14336/AD.2021.0427 34881083 PMC8612607

[B33] Lotankar S. Prabhavalkar K. S. Bhatt L. K. (2017). Biomarkers for Parkinson's disease: recent advancement. Neurosci. Bull. 33, 585–597. 10.1007/s12264-017-0183-5 28936761 PMC5636742

[B34] Manto M. Marmolino D. (2009). Cerebellar ataxias. Curr. Opin. Neurol. 22, 419–29. 10.1097/WCO.0b013e32832b9897 19421057

[B35] Mobed A. Razavi S. Ahmadalipour A. Shakouri S. K. Koohkan G. (2021). Biosensors in Parkinson's disease. Clin. Chim. Acta. 518, 51–58. 10.1016/j.cca.2021.03.009 33753044

[B36] Mullin S. Schapira A. (2015). The genetics of Parkinson's disease. Br. Med. Bull. 114, 39–52. 10.1093/bmb/ldv022 25995343

[B37] Nachman E. Verstreken P. (2022). Synaptic proteostasis in Parkinson's disease. Curr. Opin. Neurobiol. 72, 72–79. 10.1016/j.conb.2021.09.001 34653835

[B38] Opara J. Małecki A. Małecka E. Socha T. (2017). Motor assessment in Parkinson's disease. Ann. Agric. Environ. Med. 24, 411–415. 10.5604/12321966.1232774 28954481

[B39] Pan L. Meng L. He M. Zhang Z. (2021). Tau in the pathophysiology of Parkinson's disease. J. Mol. Neurosci. 71, 2179–2191. 10.1007/s12031-020-01776-5 33459970 PMC8585831

[B40] Pan-Montojo F. Reichmann H. (2014). Considerations on the role of environmental toxins in idiopathic Parkinson's disease pathophysiology. Transl. Neurodegener. 3, 10. 10.1186/2047-9158-3-10 24826210 PMC4019355

[B41] Poujois A. Woimant F. (2018). Wilson's disease: a 2017 update. Clin. Res. Hepatol. Gastroenterol. 42, 512–520. 10.1016/j.clinre.2018.03.007 29625923

[B42] Robin X. Turck N. Hainard A. Tiberti N. Lisacek F. Sanchez J. C.. (2011). pROC: an open-source package for R and S+ to analyze and compare ROC curves. BMC Bioinformatics 12, 77. 10.1186/1471-2105-12-77 21414208 PMC3068975

[B43] Ropper A. H. Samuels M. A. (1997). In Adams and Victor's Principles of Neurology.

[B44] Seong H. A. Ha H. (2019). Thr55 phosphorylation of p21 by MPK38/MELK ameliorates defects in glucose, lipid, and energy metabolism in diet-induced obese mice. Cell Death Dis. 10, 380. 10.1038/s41419-019-1616-z 31097688 PMC6522503

[B45] Su X. Federoff H. J. (2014). Immune responses in Parkinson's disease: interplay between central and peripheral immune systems. Biomed Res. Int. 2014, 275178. 10.1155/2014/275178 24822191 PMC4005076

[B46] Suykens J. A. K. Vandewalle J. J. N. P. L. (2004). Least squares support vector machine classifiers. Neural Process. Lett. 9, 293–300.11972910

[B47] Tibshirani R. (1996). Regression shrinkage and selection *via* the lasso. J. R. Stat. Soc. Series B Methodol. 58, 267–288. 10.1111/j.2517-6161.1996.tb02080.x

[B48] Vascellari S. Manzin A. (2021). Parkinson's disease: a prionopathy? Int. J. Mol. Sci. 22. 10.3390/ijms22158022 34360787 PMC8347681

[B49] Vivekanantham S. Shah S. Dewji R. Dewji A. Khatri C. Ologunde R.. (2015). Neuroinflammation in Parkinson's disease: role in neurodegeneration and tissue repair. Int. J. Neurosci. 125, 717–25. 10.3109/00207454.2014.982795 25364880

[B50] Wang C. Zhou C. Guo T. Huang P. Xu X. Zhang M.. (2022). Association between cigarette smoking and Parkinson's disease: a neuroimaging study. Ther. Adv. Neurol. Disord. 15, 17562864221092566. 10.1177/17562864221092566 35464739 PMC9019319

[B51] Wang J. He Z. Sun B. Huang W. Xiang J. Chen Z.. (2021). Pleckstrin-2 as a prognostic factor and mediator of gastric cancer progression. Gastroenterol. Res. Pract. 2021, 5527387. 10.1155/2021/5527387 34394345 PMC8360755

[B52] Wang Y. Li Y. M. Baitsch L. Huang A. Xiang Y. Tong H.. (2018). Correction: MELK is an oncogenic kinase essential for mitotic progression in basal-like breast cancer cells. eLife. 7:e36414. 10.7554/eLife.36414 29528283 PMC5847332

[B53] Yildirim M. Oztay F. Kayalar O. Tasci A. E. (2021). Effect of long noncoding RNAs on epithelial-mesenchymal transition in A549 cells and fibrotic human lungs. J. Cell. Biochem. 122, 882–896. 10.1002/jcb.29920 33847014

[B54] Yu G. Wang L. G. Han Y. He Q. Y. (2012). clusterProfiler: an R package for comparing biological themes among gene clusters. OMICS. 16, 284–7. 10.1089/omi.2011.0118 22455463 PMC3339379

[B55] Zhang J. Huang C. Liu Z. Ren S. Shen Z. Han K.. (2022). Screening of potential biomarkers in the peripheral serum for steroid-induced osteonecrosis of the femoral head based on WGCNA and machine learning algorithms. Dis. Markers. 2022, 2639470. 10.1155/2022/2639470 35154510 PMC8832155

[B56] Zheng B. Liao Z. Locascio J. J. Lesniak K. A. Roderick S. S. Watt M. L.. (2010). PGC-1α, a potential therapeutic target for early intervention in Parkinson's disease. Sci. Transl. Med. 2, 52ra73. 10.1126/scitranslmed.3001059 20926834 PMC3129986

